# Process development and validation of expanded regulatory T cells for prospective applications: an example of manufacturing a personalized advanced therapy medicinal product

**DOI:** 10.1186/s12967-021-03200-x

**Published:** 2022-01-05

**Authors:** Cristiana Lavazza, Silvia Budelli, Elisa Montelatici, Mariele Viganò, Francesca Ulbar, Lucia Catani, Marta Giulia Cannone, Sara Savelli, Elisa Groppelli, Lorenza Lazzari, Roberto M. Lemoli, Matteo Cescon, Gaetano La Manna, Rosaria Giordano, Tiziana Montemurro

**Affiliations:** 1 Department of Transfusion Medicine and Hematology, Laboratory of Regenerative Medicine, Cell Factory, Fondazione IRCCS Ca’ Granda Ospedale Maggiore Policlinico, Milan, Italy; 2 Department of Medicine and Aging Sciences, University of Chieti-Pescara, Pescara, Italy; 3 IRCCS Azienda Ospedaliero-Universitaria di Bologna, Istituto di Ematologia “Seràgnoli”, Dipartimento di Medicina Specialistica, Diagnostica E Sperimentale, Università di Bologna, Bologna, Italy; 4 Department of Internal Medicine (DiMI), Clinic of Hematology, University of Genoa, Genoa, Italy; 5 IRCCS Ospedale Policlinico S. Martino, Genoa, Italy; 6 Department of General Surgery and Transplantation, IRCCS, Azienda Ospedaliero-Universitaria di Bologna, Bologna, Italy; 7 Department of General Surgery and Transplantation, University of Bologna, Bologna, Italy; 8 Department of Experimental, Diagnostic and Specialty Medicine (DIMES)-Nephrology, Dialysis and Renal Transplant Unit, St. Orsola Hospital IRCCS, University of Bologna, Bologna, Italy

**Keywords:** ATMP, GMP process development, Process validation

## Abstract

**Background:**

A growing number of clinical trials have shown that regulatory T (T_reg_) cell transfer may have a favorable effect on the maintenance of self-tolerance and immune homeostasis in different conditions such as graft-versus-host disease (GvHD), solid organ transplantation, type 1 diabetes, and others. In this context, the availability of a robust manufacturing protocol that is able to produce a sufficient number of functional T_reg_ cells represents a fundamental prerequisite for the success of a cell therapy clinical protocol. However, extended workflow guidelines for nonprofit manufacturers are currently lacking. Despite the fact that different successful manufacturing procedures and cell products with excellent safety profiles have been reported from early clinical trials, the selection and expansion protocols for T_reg_ cells vary a lot. The objective of this study was to validate a Good Manufacturing Practice (GMP)-compliant protocol for the production of T_reg_ cells that approaches the whole process with a risk-management methodology, from process design to completion of final product development. High emphasis was given to the description of the quality control (QC) methodologies used for the in-process and release tests (sterility, endotoxin test, mycoplasma, and immunophenotype).

**Results:**

The GMP-compliant protocol defined in this work allows at least 4.11 × 10^9^ T_reg_ cells to be obtained with an average purity of 95.75 ± 4.38% and can be used in different clinical settings to exploit T_reg_ cell immunomodulatory function.

**Conclusions:**

These results could be of great use for facilities implementing GMP-compliant cell therapy protocols of these cells for different conditions aimed at restoring the T_reg_ cell number and function, which may slow the progression of certain diseases.

**Supplementary Information:**

The online version contains supplementary material available at 10.1186/s12967-021-03200-x.

## Background

Regulatory T (T_reg_) cells are an attractive type of advanced therapy medicinal product (ATMP) for adoptive cell therapy that can be used when the restoration of immunotolerance to self- or allo-antigens may prevent or even cure diseases [1–3].In murine models, expanded T_reg_ cells have been shown to be effective for the induction of long-term tolerance to bone marrow transplantation, for the prevention of graft-versus-host disease (GvHD), and for prolonging heart and skin allograft survival [4–7]. In humans receiving human leukocyte antigen-haploidentical hematopoietic stem cell transplantation for various malignancies, T_reg_ cell adoptive transfer prevents GvHD without reducing the graft-versus-leukemia effect [8]. At the time of writing the present article, the safety and the potential clinical efficacy of ex vivo-expanded autologous polyclonal T_reg_ cells are under evaluation in 48 clinical trials worldwide for indications such as end-stage kidney disease (KD), kidney or liver transplantation, type 1 diabetes, and GvHD [9]. In these conditions, T_reg_ cells could be a promising therapeutic tool to promote donor-specific transplant tolerance by exerting their immunomodulatory properties in controlling allograft rejection, for both therapeutic and preconditioning regimens, thus possibly allowing reduction and/or discontinuation of immunosuppressive drugs [10].

A critical topic for clinical applications is whether to expand T_reg_ cells from an autologous or allogeneic source. The main issue in using the latter is the risk of rejection and the resulting short survival of the donor cells, as well as possible alloimmune sensitization [11], whereas the major challenge related to an autologous product might be the difficulty in expanding T_reg_ cells and thus achieving the therapeutic dose, due to the patient’s pathology [12]. Furthermore, the manufacturing costs of an autologous product are higher than those of an off-the-shelf allogeneic product, since each batch is patient-specific [13]. However, while both autologous and allogeneic T_reg_ cells have been used in hematopoietic stem cell transplantation [12, 14], autologous cells are the preferred choice in solid organ transplantation [15].

Evidence from preclinical models suggests that the ratio between T_reg_ cells and T effector (T_eff_) cells needed to promote tolerance to organ transplantation should be much higher than the physiological level [16, 17]. Indeed, in a normal peripheral blood sample, the frequency of circulating T_reg_ cells remains constant and low (representing 2–8% of CD4^+^ T cells, < 2% leukocytes [15, 18, 19]), and a therapeutic number of T_reg_ cells can only be achieved following their in vivo or ex vivo expansion [17, 18, 20]. Several expansion protocols have been proposed to obtain a pure T_reg_ cell population that can retain its suppressive function [15, 20–23]. In general, an effective expansion protocol includes cultivation for 3–4 weeks in the presence of anti-CD3/CD28 beads, interleukin (IL)-2, and rapamycin [24, 25] to ensure a 20–200-fold increase in the number of T_reg_ cells without impairing their immunoregulatory activity [20].

In the European regulatory framework, T_reg_ cells enriched by immunoselection are not considered as a medicinal product and are regulated under the European Union Tissue and Cells Directive 2004/23/EC [26]. Instead, T_reg_ cells expanded ex vivo are classified as an ATMP, which is a substantially manipulated cellular product according to the definition in Article 2 of Regulation (EC) N. 1394/2007 of the European Parliament and of the Council of November 13, 2007 [26]. This means that expanded T_reg_ cells must be authorized by national competent regulatory authorities to be used in a clinical trial and must be approved by the European Medicines Agency to be marketed.

As part of our collaborative interinstitutional ATMP development program for adoptive cell therapy in organ transplantation, in the present paper, we describe in detail some practical issues of the whole process for T_reg_ cell expansion, starting from Good Manufacturing Practice (GMP) validation; in particular, the novel items are discussed [18, 20, 27–35]. We also pay special attention to compliance with the most recent European regulatory guidelines concerning GMP for ATMPs [36], which strongly affirm the crucial importance of a risk-based assessment to identify the potential risks associated with the manufacturing process and to control/mitigate them.

First, the practical approach we followed to design the validation process is illustrated, and then the assessment of its performance to produce GMP-compliant, clinical-scale ex vivo-expanded T_reg_ cells from patients with end-stage liver disease (LD) or KD is described. In addition, quality control (QC) method validations are explained.

## Results

### Risk assessment analysis

**Fig. 1:**
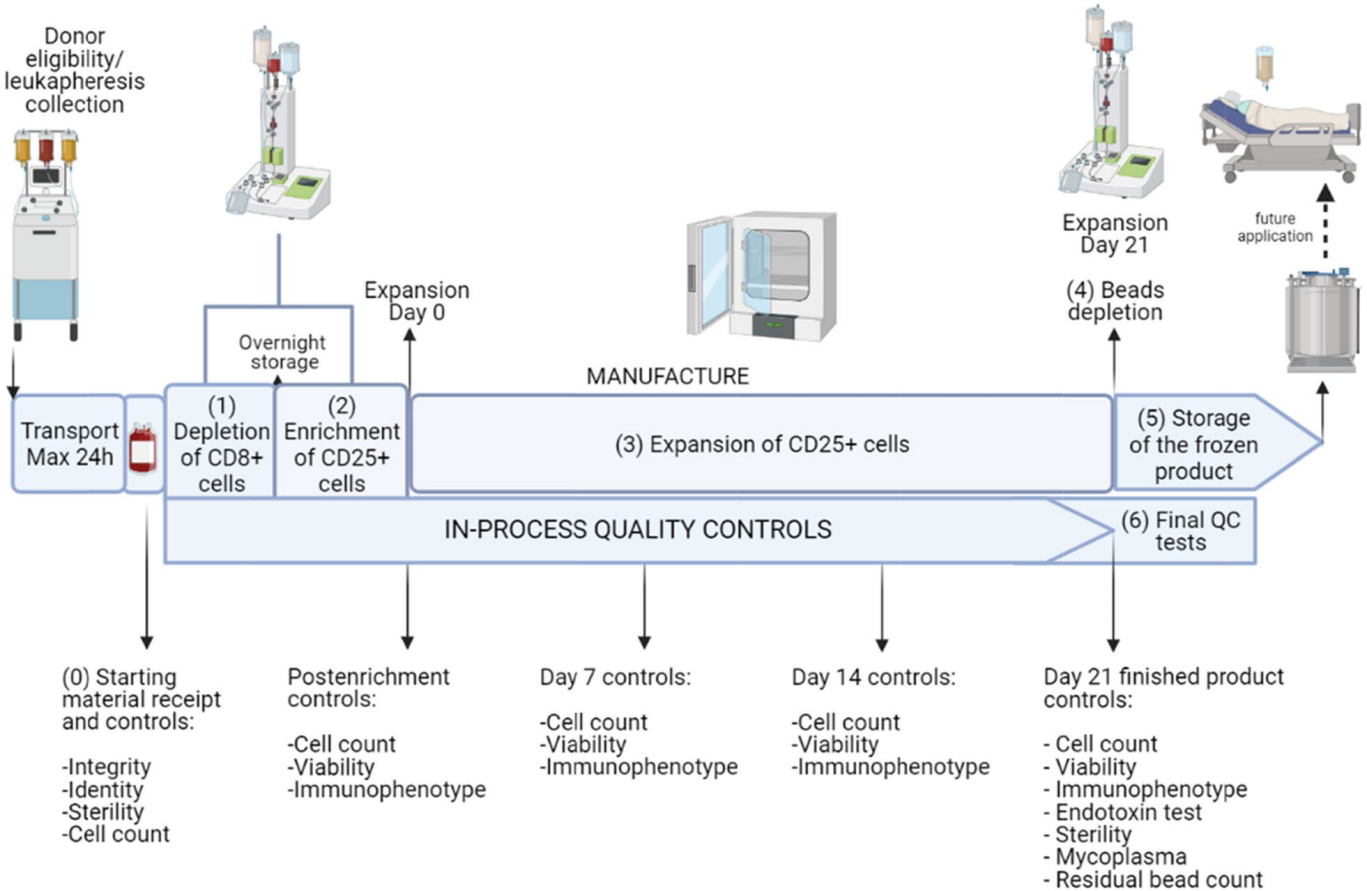
Overview of the manufacturing of expanded T_reg_ cells with in-process tests. Each step of the process validation for T_reg_ cell manufacturing was performed in accordance with GMP guidelines, with the aim of providing documented evidence that the process, performed following specific written procedures, is feasible and reproducible and produces an ATMP that meets predefined quality parameters. The process includes (0) receipt and control of the raw material after confirmation of patient eligibility, (1) depletion of CD8^+^ cells from a fresh leukapheresis unit, (2) enrichment of CD25^+^ cells from CD8-depleted fraction, (3) expansion of CD25^+^ cells for 21 days, (4) bead depletion, (5) cryopreservation of the finished product, and (6) final characterizations of the ATMP and QC testing. Multiple in-process samples (shown on the right) were taken at different stages of the process and submitted for relevant testing. Further details on GMP compliance can be found in the “Process validation design” section in the “Methods” section. Created with BioRender.com [56]

The main steps of the validation process are shown schematically in Fig. 1, while details of the operations conducted are described in the “Methods” section. Briefly, we performed (1) starting material QCs, including donor validation, leukapheresis collection, and temperature-controlled transportation; (2) determination of the number of T_reg_ cells in the starting material; (3) GMP-compliant isolation of CD8^−^ CD25^+^ cells, and verification of selection efficiency by flow cytometry; (4) large-scale expansion of T_reg_ cells, by performing all in-process controls and release tests on the finished product; (5) functional testing of T_reg_ cells (in vitro suppression test); and (6) cryopreservation and thawing, and subsequent evaluation of the expansion ability of the thawed T_reg_ cells.

For each process step, all potential hazards and accidental events that could cause failures were identified. By performing a preliminary hazard analysis (PHA), a risk score was assigned to each hazardous situation according to the criticality matrix shown in Additional file 1: Table S1. All listed points were taken into consideration for effective risk evaluation in order to select an appropriate action plan. A total of nine hazardous topics were identified, among which seven were quoted as other than acceptable (3 tolerable, 4 unacceptable) without the implementation of strategies for risk control. The highest risks identified were those associated with the environment and documentation. Based on the analysis performed, the point-by-point mitigation plan described in Table 1 was set. All identified risks could be reduced by implementing the mitigation measures. Thus, the scenarios with unacceptable risk declined from 44% (4 out of 9 categories) to 0%, and those with acceptable risk increased from 22% (2 out of 9 categories) to 100%. No one risk remained unacceptable.

**Table 1:**
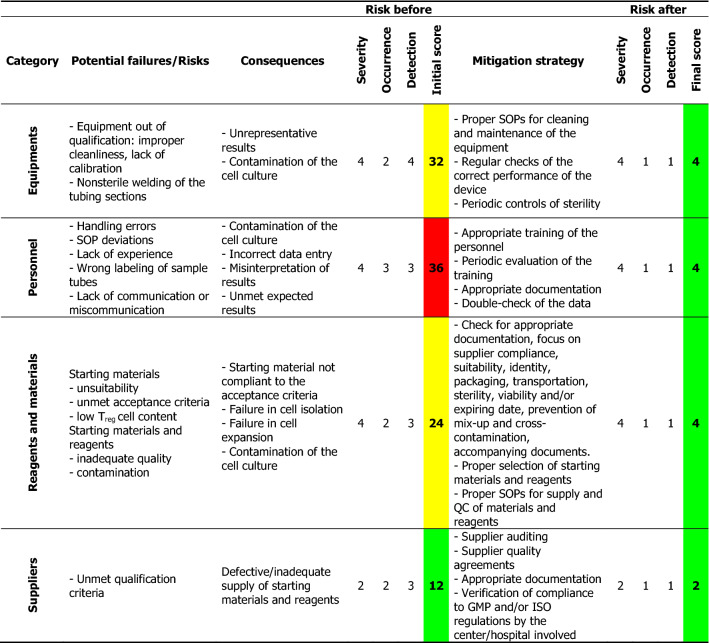

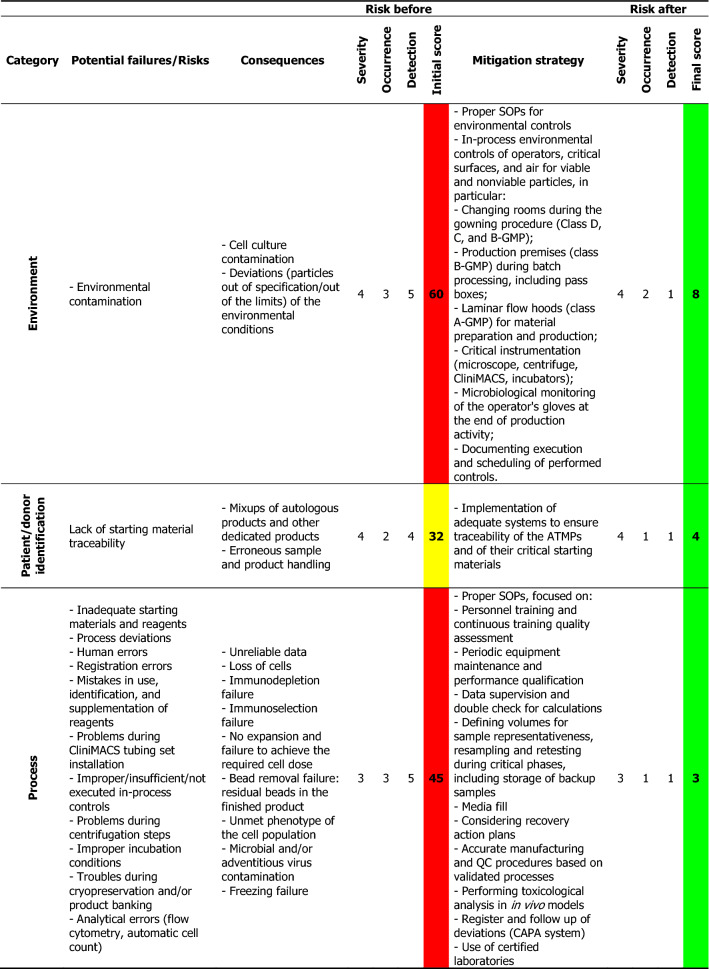

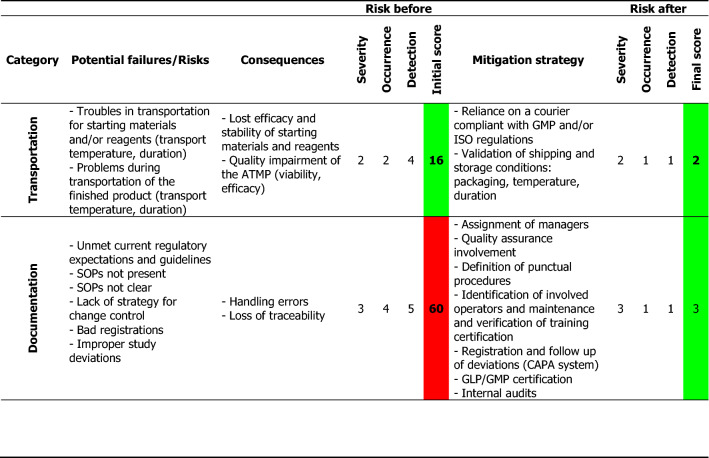
The potential failures, scores, and proposed mitigation approaches identified by PHA

The risk score was calculated as [Severity × Likelihood of recurrence], where Likelihood = (Occurrence × Detection), the Severity ranking was assigned based on the severity of the consequences of failure, the Occurrence ranking rates the probability of a failure occurring, and the Detection ranking indicates the chances of detecting a failure before it occurs using customized ranking scales as a guide (Additional file 1: Table S1)

*SOP* standard operating procedure, *QC* quality control, *ISO* International Organization for Standardization, *GMP* good manufacturing practice, *CAPA* corrective and preventive actions, *GLP* good laboratory practice

### Patient samples and starting material QCs

The patient characteristics are described in Table 2. All patients met the requirements of the Italian Legislative Decree of January 25, 2010, n. 16; negativity for HIV, HBV, and HCV active infection was also confirmed by TRI-NAT on predonation samples within 24 h of the harvesting procedure.

**Table 2:** Patients’ characteristics and inclusion criteria

Parameter	Inclusion criteria	Patients [validation run]			
		KD1 [#01]	KD2 [#02]	LD1 [#03]	LD2 [#04]
Age	≥ 18 years	47 years	62 years	61 years	60 years
Gender	Unconcerned	Male	Female	Male	Male
Medical condition	End-stage disease	End-stage kidney disease	End-stage kidney disease	End-stage liver disease (alcoholic cirrhosis)	End-liver liver disease
HIV1/2 (Ag/Abs)^§^	Negative	Negative (4/4 patients)			
Anti-HCV Abs^§^	Negative	Negative (4/4 patients)			
HBs Ag^§^	Negative	Negative (4/4 patients)			
Anti-HBc Abs^§^	Negative	Negative (4/4 patients)			
Anti-VDRL Abs^§^	Negative	Negative (4/4 patients)			
Informed consent	Signed	Signed (4/4 patients)			

*KD* patient with kidney disease, *LD* patient with liver disease, *Ag* antigen, *Abs* antibodies

^§^Chemiluminescent microparticle immunoassay (CMIA)

The specifications for the starting material are shown in Table 3. On average, the leukapheresis products (n = 4, mean volume: 60.3 ± 7.4 mL) were shipped to our facility within 03:03 ± 00:38 h from departure and within 04:57 ± 01:20 h from the end of collection. The mean transportation temperature was 2.9 ± 2.5 °C.

**Table 3:** Predefined and final specification for the starting material

Pre-acceptance controls	Pre-defined specification	Validation run					Final specification
		#01	#02		#03	#04	
Accompanying documents	Present	Present (4/4 batches)					C
Bag identificative	Anonym, univocal code	Anonym, univocal code (4/4 batches)					C
Volume harvested	To be set	58 mL	71 mL	54 mL		58 mL	≥ 50 mL
Transportation mean temperature [range]	To be set (transport at controlled temperature in refrigerated container with ice packs and validated data logger)	Not available	0.4 °C [− 3.9–13.1]	5.3 °C [4.0–24.6]		3.1 °C [2.2–6.7]	2–8 °C
Time from harvesting	Up to 12 h from the completion of collection to receipt at the manipulation site	06h53	03h59	04h51		04h08	≤ 12 h
State of the collection bag	Intact and appropriately sealed	Intact and appropriately sealed (4/4 batches)					C
BacT/ALERT aerobic	Sterile	Sterile (4/4 batches)					C
BacT/ALERT anaerobic	Sterile	Sterile (4/4 batches)					C

*C* compliant with predefined specifications

All of the leukapheresis products were approved as a starting material for the manufacturing process according to the predefined criteria listed in Table 3, despite the fact that the transport temperature of the starting material was not available for the first run due to a failed temperature registration. Indeed, this failure was managed as an unplanned deviation according to GMP with an appropriate investigation. During the deviation management, several aspects were investigated. Among these, the shipping conditions were reviewed: it was verified that the delivery of the starting material was carried out with dedicated transport, by a qualified courier working in accordance with Good Distribution Practice [37]; in addition, the packaging conformity of the transport box was assessed. Furthermore, the starting material was checked for compliance with all of the other required specifications listed in Table 3. Finally, the CD45^+^ cell viability in the starting material was ≥ 90% (Table 4). Consequently, the donor#1 starting material was determined to be eligible for validation.

**Table 4:** Characterization and enumeration of T_reg_ cells before and after enrichment procedures

	Parameter	Validation run				KD patients	LD patients
		#01 [KD1]	#02 [KD2]	#03 [LD1]	#04 [LD2]	Mean ± SD	Mean ± SD
Starting material	Viable CD45^+^ cells (%)	99.4	99.8	99.8	99.9	99.6 ± 0.3	99.9 ± 0.1
	Apheresis’ processed volume (mL)	54.4	69.2	50.8	54.8	61.8 ± 10.5	52.8 ± 2.8
	WBC concentration (× 10^6^/mL)	194.0	129.5	116.0	44.5	161.8 ± 45.6	80.3 ± 50.6
	T_reg_ cells concentration (× 10^6^/mL)	6.32	3.04	1.98	0.82	4.7 ± 2.3	1.4 ± 0.8
	T_reg_ cell absolute numbers (× 10^6^)	343.6	210.3	100.6	44.9	277.0 ± 94.3	72.8 ± 39.4
	T_reg_ cells (%)	2.6	1.8	1.3	1.6	2.2 ± 0.6	1.5 ± 0.2
	CD127^−^ T_reg_ cells (%)	na	1.7	2.0	2.1	1.7 na	2.1 ± 0.1
	CD45^+^CD8^+^ cells (%)	27.2	34.3	22.2	25.0	30.8 ± 5.0	23.6 ± 2.0
Post-enrichment fraction	T_reg_ cells absolute numbers (× 10^6^)	116.7	91.1	54.4	41.1	103.8 ± 18.0	47.8 ± 9.4
	T_reg_ cells purity (%)	84.9	81.3	53.9	70.6	83.1 ± 2.5	62.3 ± 11.8
	CD127^−^ T_reg_ cells (%)	65.6	70.1	47.1	66.8	67.9 ± 3.2	57.0 ± 13.9
	FoxP3^+^ T_reg_ cells (%)	84.9	54.1	47.0	59.9	69.5 ± 21.8	53.5 ± 9.1
	CD127^−^ FoxP3^+^ T_reg_ cells (%)	65.4	51.6	45.3	58.9	58.5 ± 9.8	52.1 ± 9.6
	CD45^+^ CD8^+^ cells (%)	0.8	0.1	0.6	0.1	0.5 ± 0.5	0.4 ± 0.4
	Recovery (%)	33.9	43.3	54.1	91.5	38.6 ± 6.6	72.8 ± 26.5

All data are presented as viable cells

*SD* standard deviation, *na* not available

As shown in Fig. 1, sterility assessment was immediately performed as the first manipulation step by direct inoculation of both aerobic and anaerobic microorganisms. The data collected during the validation runs were used to set the final limits and specifications for the starting material to guarantee a final product with predefined quality characteristics.

### Determination of the number of T_reg_ cells in the starting material

**Fig. 2:**
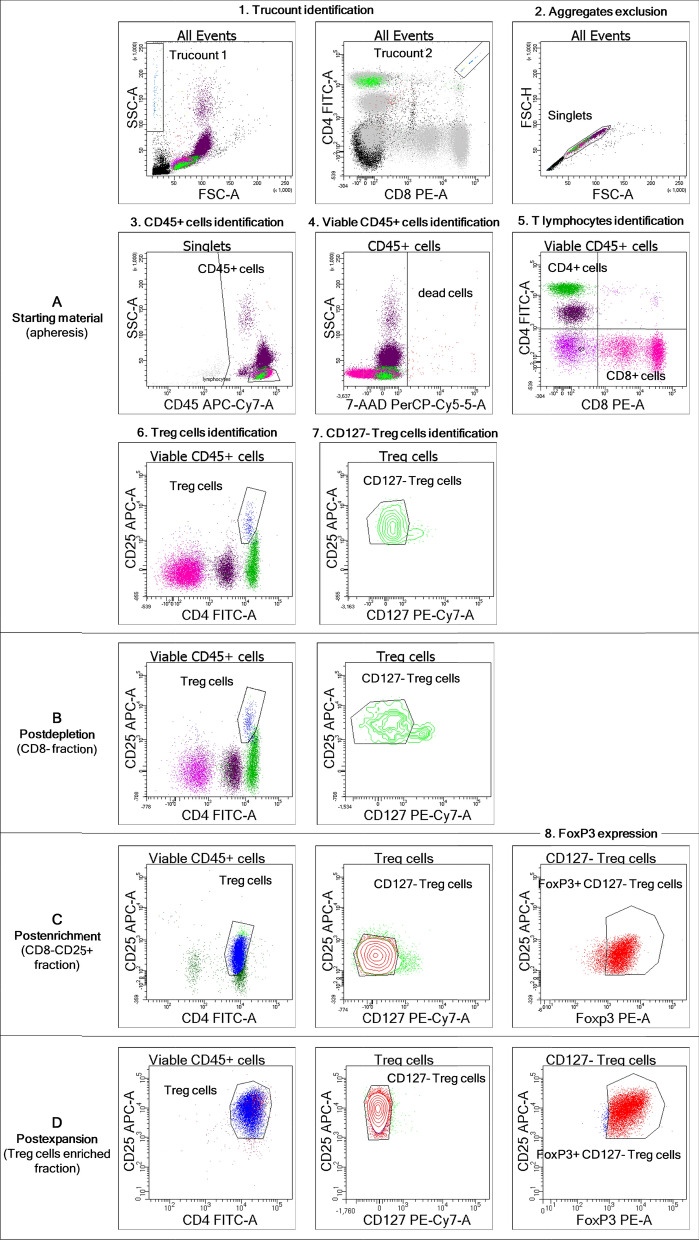
Flow cytometric analysis of T_reg_ cells along the manufacturing process. Whole blood (starting material) or in-process samples (postdepletion, postenrichment, and postexpansion) were stained with fluorochrome-conjugated antibodies against surface markers CD45, CD4, CD25, and CD127 in TruCOUNT tubes and analyzed according to the lyse-no wash method. **A** gating strategy for T_reg_ cell enumeration in whole apheretic samples. **A** (step 6) to **D** T_reg_ cell purity quantification along the manufacturing process: starting material (**A** step 6), postdepletion (**B**), postenrichment (**C**), and postexpansion (**D**) fractions. In detail: Trucounts for absolute cell counts were identified by the intersection of events gated based on standard light scattering characteristics and those gated based on fluorescence, as shown in a PE vs. FITC plot with both plots showing all acquired events (step 1). Cell aggregates and debris were excluded on an FSC-A vs. FSC-H dot plot (step 2), followed by CD45^+^ cell identification on an APC-H7 vs. SSC-A dot plot (step 3). Among CD45^+^ cells, viable cells were gated based on negativity for 7-AAD staining (step 4). Within viable CD45^+^ cells, cells expressing CD4 or CD8 were detected in a PE vs. FITC plot (step 5), whereas CD4^+^CD25^+^ T_reg_ cells were identified in a FITC vs. APC plot (step 6). Finally, among T_reg_ cells, CD127^−^ cells were gated on a PE-Cy7 vs. APC dot plot (step 7). For samples with a higher content of T_reg_ cells (postenrichment and postexpansion), twin samples were stained for a surface marker in FACS tubes and then fixed and permeabilized for intracytoplasmic staining of FoxP3, which was detected among CD127^−^ T_reg_ cells on a PE vs. APC dot plot (step 8). Representative images of samples from patient KD2 are shown. The negative control for FoxP3 staining is shown in Additional file 1: Fig. S1

We initially characterized and counted the T_reg_ cells contained in the starting material, as described in the “Flow cytometry” paragraph of the “Methods” section. The T_reg_ cell signature is defined by a high expression of the surface CD25 molecule among CD4^+^ cells; T_reg_ cells also poorly express or are negative for CD127 in the presence of the transcription factor FoxP3 [38]. To enumerate the T_reg_ cells, we standardized an absolute cell count method for T_reg_ cell enumeration based on Trucount tubes by adopting the gating strategy shown in Fig. 2A. The detailed results are reported in Table 4. By considering the median values for an apheresis product volume of 54.6 mL (range 50.8–69.2) with a whole blood cell (WBC) content ranging from 2.4 × 10^9^ to 10.6 × 10^9^, the absolute number of viable T_reg_ cells was 155.5 × 10^6^ cells (range 44.9 × 10^6^–343.6 × 10^6^). Overall, T_reg_ cells accounted for 1.8 ± 0.6% of the CD45^+^ cells, on average, and they were almost all negative for CD127.

### GMP-compliant isolation of CD8^−^CD25^+^ cells

T_reg_ cells were isolated by a two-step immunoselection procedure, as shown in Fig. 1 and detailed in “GMP-grade isolation of T_reg_ cells” in the “Methods” section. After the depletion procedures, there were few contaminating CD8^+^ cells (median: 0.1%, range 0–0.4%), with a median WBC recovery of 65% (range 50–70%) of the starting material, and this value was similar for both the KD and LD patients (58 ± 11% and 67 ± 5%, respectively). Consequently, the T_reg_ cell content was also decreased, compared to the starting material, especially for the KD patient samples (157 ± 65 × 10^6^ preselection vs. 277 ± 94 × 10^6^ postselection T_reg_ cell number) that had a T_reg_ cell recovery of 56 ± 4% vs. 94 ± 8% for the LD patient samples (67 ± 31 × 10^6^ vs. 73 ± 39 × 10^6^). As expected, the CD8^+^ cell loss did not significantly affect the T_reg_ cell concentration in the postdepletion fraction (median: 2.5%, range 2.1–2.9%), compared to that of the starting material (median: 1.7%, range 1.3–2.6%).

An increase of the T_reg_ cell purity in the cell product throughout the manufacturing process is illustrated in Fig. 2 and Table 4. After CD25^+^ cell enrichment, positive fractions (CD8^−^ CD25^+^ cells) contained a median of 94 × 10^6^ total nucleated cells (range 59 × 10^6^–178 × 10^6^), with a median T_reg_ cell purity of 76% (range 54–85%). A decrease of CD25, according to the mean fluorescence intensity, was detected in the postenrichment samples due to anti-CD25 antibody binding during immunoselection. Concerning the T_reg_ cell enrichment efficiency, the median recovery was 49% (range 34–92%) from the leukapheresis product (Table 4). Overall, the two-step immunoselection procedure allowed the recovery of 73 × 10^6^ T_reg_ cells (median) from an apheresis volume of about 55 mL.

On average, more than 61% of the isolated T_reg_ cells expressed FoxP3 or CD127 to a low extent or not at all; in addition, more than 55% of the isolated T_reg_ cells presented both signatures (Table 4). Contaminating CD8^+^, CD19^+^, or CD56^+^ cells did not exceed 4% in the postenrichment fraction.

### Clinical scale expansion capacity of T_reg_ cells ex vivo

In runs #01, #02, and #03, 40 × 10^6^ freshly isolated CD8^−^CD25^+^ cells (29 ± 7 × 10^6^ T_reg_ cells) were continuously expanded in gas-permeable culture bags in the presence of allogeneic heat-inactivated plasma, rapamycin, IL-2, and expansion beads by performing two subcultures, as detailed in Fig. 1. After expansion for 21 days, bead immunodepletion was performed, according to the manufacturer’s instructions, to obtain the target fraction; aliquots of the target fraction were then subjected to QC analysis or cryopreserved for additional experiments, as detailed in “QCs: in-process and release tests” in the “Methods” section.

**Fig. 3:**
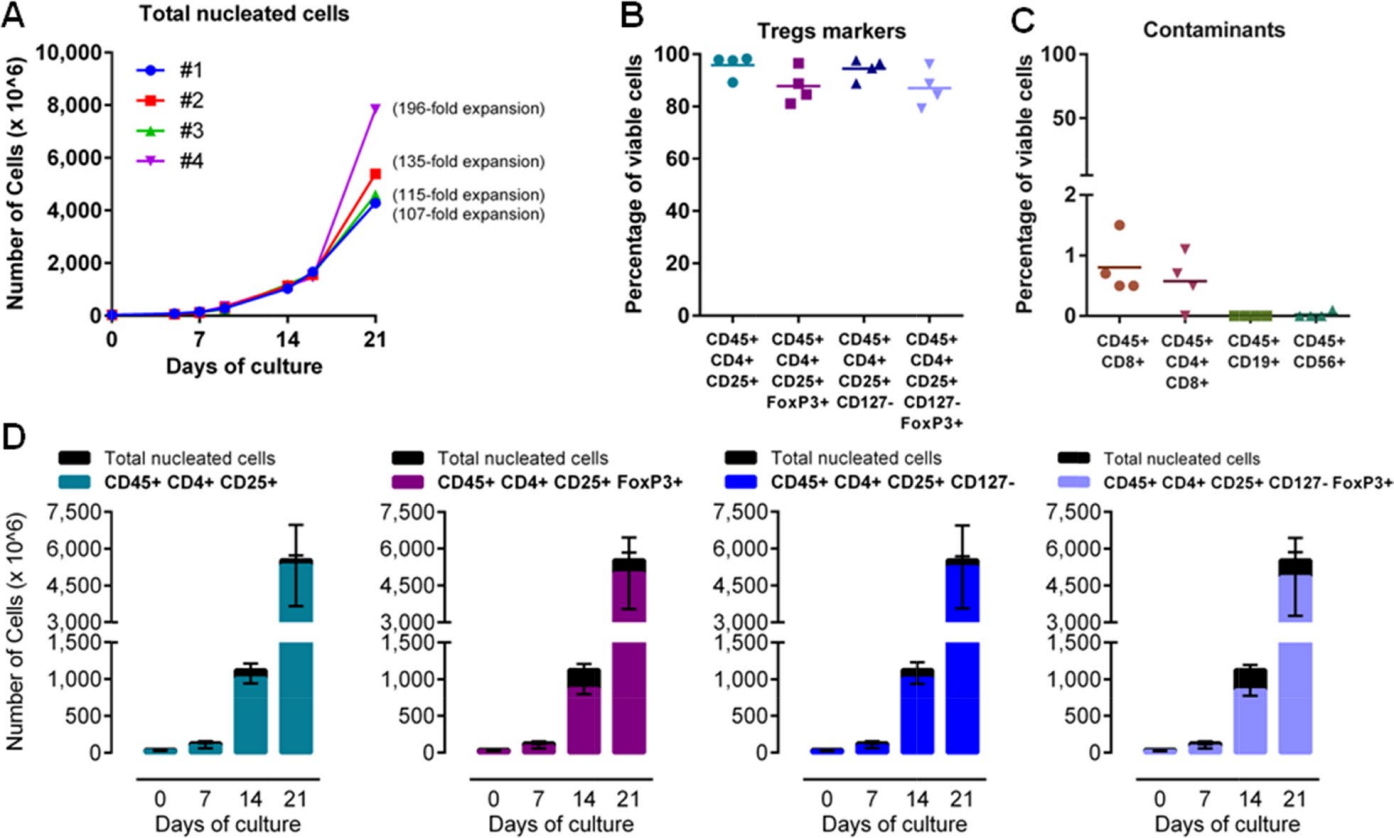
Ex vivo T_reg_ cell expansion. Isolated CD8^−^CD25^+^ cells derived from KD and LD patients were expanded in vitro in gas-permeable culture bags for 3 weeks in complete medium. **A** Kinetics of proliferation at a clinical scale. **B** Day-21 expression of discriminating markers CD127 and FoxP3; **C** frequency of contaminating populations. T_reg_ expanded after thawing (n = 1, run #04 LD2, diamond shape) displayed no relevant differences as compared to freshly expanded T_reg_ (all other symbols). **D** The mean achievable absolute number of cells in relevant subpopulations. Frequencies were expressed as percentage of viable cells; absolute numbers were normalized over seeded cells

As shown in Fig. 3, a median of 4.6 × 10^9^ expanded cells (range 4.3 × 10^9^–5.4 × 10^9^) with a T_reg_ cell purity of 95 ± 5% were obtained. On average, the T_reg_ cells achieved 159 ± 33-fold expansion over seeding (median: 162-fold; range 124–191, n = 3), yielding a median total dose of 4.2 × 10^9^ T_reg_ cells (range 4.1 × 10^9^–5.3 × 10^9^) (Fig. 3). FoxP3^+^ and CD127^−^FoxP3^+^ T_reg_ cells accounted for 91 ± 6% and 87 ± 9% of the expanded cells, respectively. Contaminating CD8^+^ T cells represented less than 1.5% of the total (median: 0.7%, range 0–1.1), resulting in a maximum number of 11 CD8^+^ T cells for every billion expanded T_reg_ cells (validation run #02, KD2), with a median of 7 CD8^+^ T cells for every billion expanded TNCs (range 5–15). After enrichment, there was a negligible amount of CD56^+^ cells (median: 0%, range 0–0.1%), whereas contaminating CD19^+^ B cells were undetectable.

On day 21, the magnetic immunodepletion allowed the removal of 99.99% of the beads, compared to the original fraction. Indeed, starting from 2.9 ± 0.7 × 10^6^ beads, for every 30 × 10^6^ cells in the original fraction, an average of 319 ± 142 beads for every 30 × 10^6^ cells in the target fraction was found (p < 0.008 paired t test). Further details on expansion are provided in Table 5. A cryopreserved aliquot was prepared as a retention sample, on which a test for information only was performed, including the determination of cell viability after thawing (data not shown).

**Table 5:** Expansion of T_reg_ cells

	Culture day	Freshly expanded			Recovered after thawing
		#01 [KD1]	#02 [KD2]	#03 [LD1]	#04 [LD2]
Total cells fold expansion	0	1.0	1.0	1.0	1.0
	7	3.8	2.9	3.2	3.5
	14	26.0	28.6	30.0	28.2
	21	107.1	134.9	115.2	196.1
T_reg_ cells fold expansion	0	1.0	1.0	1.0	1.0
	7	Nd	2.9	3.0	4.6
	14	27.9	32.0	44.8	39.0
	21	124.0	162.0	190.6	271.9
Number of T_reg_ cells (normalized over seeded cells) (× 10^6^)	0	34.0	32.5	21.6	28.2
	7	Nd	94.1	63.8	131.0
	14	948.2	1041.2	965.3	1102.4
	21	4211.7	5268.3	4110.2	7678.8

On day 0, 7, 14, 21 expanding cells were phenotyped for T_reg_ markers and enumerated by flow cytometry with a single platform (Trucount)

### Expansion ability of thawed T_reg_ cells

Freezing and banking of expanded T_reg_ cells will guarantee the clinical utility of the expansion strategies in different clinical settings (e.g., multiple treatment procedures or allogeneic use). To test if the freezing of expanding T_reg_ cells could affect their expansion ability after thawing, a fourth expansion run (#04, LD2) with a stop-and-thawing procedure was performed. We focused our attention on day 14, as this is the point in the process where the expansion curve accelerates (Fig. 3A). Specifically, we stopped the cell culture on day 14, cryopreserved the expanding cells, and restarted the expansion after thawing by seeding the cells in a culture bag as for the standard culture at the same time point, without an additional post-thaw period of restimulation. We recovered 85% of the cryopreserved cells, with 89.1% cell viability, as reported previously; this finding indicated that the 14-day-expanding T_reg_ cells successfully survived cryopreservation. The T_reg_ cell markers on the thawed cells were retained and were similar to those on the prefrozen cells (data not shown), as described previously [7]. The thawed T_reg_ cells were expanded during the last expansion week (from day 14 to day 21) in a culture bag as performed for the other runs. The fold-expansion of cells recovered after thawing was comparable to that of continuously cultured ones; we obtained a cumulative 272-fold increase in the number of T_reg_ cells, which was not different from that seen with fresh T_reg_ cells (mean 159 ± 33, n = 3, Fig. 3 and Table 5). Additionally, 21-day-expanded cells from run #04 (LD2) retained their immunophenotypic and functional characteristics and were comparable to those of the other runs (Fig. 3).

### Validation of compendial analytical method and results

#### Sterility test

The growth of microorganisms was observed in the presence and absence of the product for all validation batches, indicating that the freezing of the product and the final cryopreserved product composition itself do not affect the growth of microorganisms. Specificity (no false positives) was assessed by evaluating microorganism growth by incubating the culture media alone. The detection limit (sensitivity) was 1–10 CFU, as expected, i.e., there was a similar growth to that obtained for the positive controls with both concentrations of strains used. Intermediate precision was confirmed by analyzing the results by two different operators on two different days. All validation samples were determined to be sterile for both aerobic and anaerobic microorganisms.

#### Bacterial endotoxins

Confirmation of the lysate sensitivity, expressed in EU/mL, was carried out by performing four replicates of the gel clot analysis for four concentrations of control standard endotoxin (CSE), equivalent to 2λ, λ, 0.5λ, and 0.25λ, and a negative control (only water for the bacterial endotoxin test). The sensitivity found for the lysate was 0.03 EU/mL. In our conditions, 60 mL was the maximum volume infused for a mean body weight of 70 kg, so M = 0.86 mL/kg, K = 5 EU/kg [39], EL = 5.8 EU/mL, and MVD = 193.33 for λ = 0.03 EU/mL.

The preliminary study on product batches diluted 1:5, 1:10, and 1:100 showed that only the 1:10 and 1:100 dilutions did not interfere with clot formation, in accordance with the validity criterion. The endpoint in the final validation for the other two product batches was met by choosing the minimum dilution of the noninterfering product (1:10). The endpoint was determined to be 0.03 EU/mL, confirming that the product at this dilution does not interfere with clot formation. The sensitivity of the test calculated for this specific product was calculated according to the formula: sensitivity × chosen dilution (0.03 EU/mL × 10) = 0.3 EU/mL. Thus, the specification for the endotoxin test can be fixed, considering the range between the sensitivity of the test (0.3 EU/mL) and the calculated endotoxin limit (5.8 EU/mL).

The product, under the experimental conditions used, does not contain interfering factors if the sensitivity of the lysate determined with the diluted product in the presence of CSE is not less than 0.5λ and is not greater than 2λ.

#### Mycoplasma test

The growth of *Mycoplasma pneumoniae* and *M. hominis* at a concentration of 10–100 UFC/mL was found from day 3 to day 20 in liquid media and from days 2–4 to days 19–21 in solid media from the inoculation at different time points both in the presence and in the absence of the product. For each microorganism inoculated, there was no difference greater than 5 CFU/mL, both in the presence and in the absence of the product.

Four out of four batches produced by large-scale GMP-compliant expansion were fully compliant according to the predefined acceptance criteria for all microbial contamination methods, as shown in Table 6, which summarizes the validation data.

**Table 6:** Release criteria for the finished product and quality controls performed on the target fraction during the validation process

Parameter	Validation run				*Finished product specification*
	#01 [KD1]	#02 [KD2]	#03 [LD1]	#04 [LD2]	
TNC viability	nd	97.9%	98.3%	98.9%	≥ 90%
Purity	98.3%	97.6%	89.2%	97.9%	> 80%
CD127^−^ T_reg_ cells (%)	94.7%	96.5%	88.9%	97.6%	> 80%
FoxP3^+^T_reg_ cells (%)	91.1%	96.5%	84.6%	88.8%	> 80%
CD127^−^FoxP3^+^ (%)	79.2%	96.1%	84.4%	88.6%	> 80%
CD45^+^CD19^+^ (%)	0.0%	0.0%	0.0%	0.0%	< 2%
CD45^+^CD56^+^ (%)	0.1%	0.0%	0.0%	0.0%	< 2%
CD45^+^CD8^+^ (%)	0.5%	1.5%	0.7%	0.5%	< 2%
Residual beads count^§^	na	405	397	156	< 1000
Microbial growth (sterility)^#^	Sterile (4/4 batches)				Sterile (no growth)
Mycoplasma	No growth				No growth
Endotoxin	< 0.3 EU/mL (4/4 batches)				≤ 0.3 EU/mL

All data refers to viable cells

*na* not available

^§^Absolute number/30 × 10^6^ TNC target fraction

^#^After 10 days

### Release specifications of the T_reg_ cell drug product, batch analyses, and stability

Every step of the manufacturing process was documented and performed according to the batch records and standard procedures. Based on characterization of the finished product obtained during our large-scale validation runs, the release specification for the ATMP was defined (Table 6). Thanks to compliance with the predefined criteria and low inter-run variability of the results, quite stringent product definition criteria were set (Table 6).

### Functional testing of T_reg_ cells (in vitro suppression assay)

**Fig. 4:**
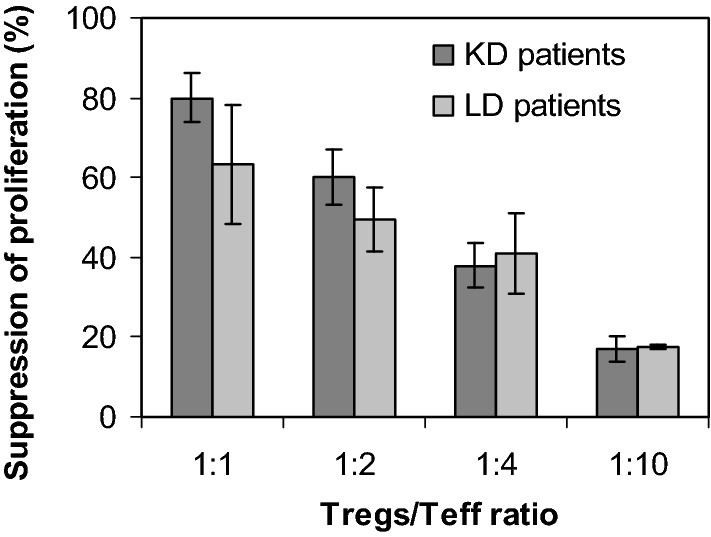
Functional properties of thawed T_reg_ from KD (dark gray) and LD (grey) patients. The suppressive function of expanded T_reg_ was analyzed by measuring the proliferation of CFSE-stained CD8^−^CD25^−^ cells primed with anti-CD3/CD28 beads in co-culture with expanded T_reg_ cells for 5 days. Percentages of suppression are presented as mean values; bars represent standard deviations of 2 independent experiments

Day-21 GMP-expanded T_reg_ cells displayed an increased T_reg_ phenotype, suggesting an increased suppressive function compared to freshly isolated T_reg_ cells [7]. As a decrease of expanded T_reg_ cell function after cryopreservation has been reported [40–42], the immunosuppressive capacity of T_reg_ cells was tested in runs #01 to #04 after cryopreservation and thawing. Similar to previous data [7, 32], the thawed T_reg_ cells effectively inhibited T_eff_ cells in a dose-dependent manner (Fig. 4). The mean inhibitory activity was recorded at a T_reg_ cell/T_eff_ cell ratio of 1:2 (n = 4, 53.0 ± 8.2% inhibition); at a 1:1 T_reg_ cell/T_eff_ cell ratio, the T_reg_ cells showed the highest suppressive capacity (69.0 ± 14.2%), and their activities were comparable, irrespective of the patient pathology and regardless of whether their expansion was performed in a one-step or in a two-step manner (median: 66.5% vs. 74%, for continuous and stop-and-thawed culture, respectively, Fig. 4).

## Discussion

Herein, we describe the process validation for a safe, reproducible, and flexible GMP manufacturing process for isolation, expansion, and cryopreservation of expanded T_reg_ cells from patients affected by end-stage KD or LD.

The aim of this work was to describe the steps followed for the validation of a manufacturing process adaptable to small-size and/or academic pharmaceutical plants to obtain a final product available for clinical applications in which immunomodulation is required. This objective is particularly valuable when considering that T_reg_ cells can positively affect or even counteract the evolution of severe diseases and/or reduce the need for immunosuppressive therapy, with beneficial results on therapy-related side effects and healthcare costs.

In a healthy human adult subject, T_reg_ cells represent up to 5–10% of the total circulating T cells [3], meaning that at most 200 × 10^6^ T_reg_ cells from leukapheresis can be isolated [30]. Therefore, ex vivo expansion is essential to obtain a sufficient number of T_reg_ cells in order to impact the immune response to an allograft after transplantation in humans. In this regard, various groups have recently demonstrated a beneficial T_reg_ cell-dose dependent effect on alloreactivity suppression for tolerance induction after liver transplantation [43, 44].

Due to the extent of cell manipulation, expanded T_reg_ cells are classified as an ATMP according to the European regulatory framework [26]. As recommended by the European Medicines Agency regarding the inclusion of the quality-by-design approach for the development of an investigational medicinal product, we performed a process risk assessment in which all sources of variability potentially affecting a process are identified, explained, and managed by appropriate measures. For this purpose, we used PHA to identify, classify, and describe possible risks, dangerous situations and events that could cause failure, their origin, and possible consequences (risks) as well as to estimate the probability of occurrence for a given potential failure.

We set up a detailed strategy (Table 1) by which we could identify different potential failures according to the category (e.g., equipment, personnel, reagents, suppliers, environment, and the patient or the process itself) and their possible consequences, and we gave a severity score to each of them according to the respective severity of occurrence (i.e., a higher score indicated a higher risk). Mitigation strategies were proposed for each encountered risk, leading to a significant reduction of the possible final score. As mentioned, this method defines the corrective actions to modify, control, or delete dangerous situations as well as measures the safety and reliability of the manufacturing process. Furthermore, it could be of great use as a guide for the implementation of corrective actions from the very beginning (from the proper use of standard operating procedures, to the appropriate training of the personnel, traceability systems, etc.).

We also provided a practical example of how we managed an unexpected deviation that actually occurred for the transport of starting material. Indeed, despite the implementation of control strategies, a deviation could still happen. According to GMP, an investigation file must be immediately opened to classify the severity of the failure and to identify the causes, with the aim of preventing or limiting the possible negative impact to the process. The investigations and corrective actions implemented must be recorded in dedicated documentation that must become an addendum to the batch record and must be evaluated before the validation process is approved. Indeed, a possible deviation can always occur, not only in the validation phases of a process but also during a clinical trial, and it should be properly managed in time and with an appropriate approach according to GMP, rather than leading to a possible rejection of a clinical sample.

To monitor the progression of such a long manufacturing process, besides the identification of the critical quality attributes, establishment of the appropriate assays at different steps of the process to ensure the quality of intermediate and finished products is critical. In our opinion and based on our experience, even if the validation of a production process and the QC methods are only suggested in the last revision of the regulations concerning ATMPs [36], even the most accurate risk analysis cannot completely replace a validation step, especially for the analytical methods. Therefore, the release QC validation should be performed according to the official Pharmacopoeia whenever possible (e.g., for compendial methods).

The results of the validation work for the different parameters indicated reliable results for viability and purity, with positive consequences on the robustness of our manufacturing process and on its ability to produce a high-quality ATMP.

A critical step to consider before clinical use is the depletion of immunomagnetic beads from the expansion product. The efficacy and safety of anti-CD3/CD28 expansion beads in vivo are actually not well known. To ensure safety of the finished product on day 21, bead removal from expanded T_reg_ cells was performed by magnetic immunodepletion, according to the manufacturer’s instructions. For QC of residual bead enumeration, we performed a specific validation in agreement with GMP, as the procedure proposed by the manufacturer is not intended for drug development but for research use only [45].

Therefore, we set and validated a method for bead enumeration in the finished product based on the raw count in the Bürker chamber (see the “Materials and Methods” section for details) and the use of a reduced number of cells for QC, compared to what is required by the protocol proposed by the manufacturer. We decided not to follow the manufacturer-suggested procedure because of several critical issues: (1) most cytometers do not provide an absolute event count without the addition of counting beads, which was difficult in our case, since the reference beads were difficult to distinguish from the expansion beads; (2) the number of “wasted” cells (original fraction corresponding to 5–20 × 10^4^ MACS GMP ExpAct T_reg_ Beads and 1 × 10^8^ cells for the target fraction, both in triplicate) required for bead enumeration affected the clinical dosage; (3) repeated centrifugations and discarding of the supernatant would invariably lead to unpredictable and unstandardized bead loss, and the consistency might vary depending on the different sample matrixes (e.g., original fraction, target fraction, and control), thus affecting the method accuracy.

Due to a robust and consistent expansion capability, regardless of the number of circulating T_reg_ cells, the process we validated will allow patients to be enrolled in clinical trials. Indeed, we demonstrated that despite the fact that the cellularity of the apheresis product may vary due to the harvest procedure, the T_reg_ cell content in the starting material does not influence the isolation efficiency.

With the manufacturing process we described, we were able to obtain a clinically relevant cell dose of 79 ± 23 × 10^6^ T_reg_ cells/kg for a mean body weight of 70 kg; of note, a target cell dose for a clinical trial is generally 1–10 × 10^6^ T_reg_ cells/kg [3]. Moreover, ex vivo expansion also allowed us to obtain a purer product than that obtained by direct isolation. In our experience, large-scale T_reg_ cell selection using the CliniMACS isolation system from leukapheresis yielded a CD4^+^ CD25^+^ T-cell purity of 55% (range 42.6–62%), the majority of which expressed FoxP3, in keeping with the reported data from healthy subjects [40, 46, 47].In response to recent studies showing the negative effect of cryopreservation on T_reg_ cell function [41, 42], we have previously demonstrated that expanded T_reg_ cells after thawing can effectively prevent the onset of xenogeneic GvHD as well as improve acute GvHD and survival in a mouse model of GvHD using immunosuppressed mice (i.e., NOD-SCID-gamma knockout mice) [7]. Herein, the in vitro data reported also confirmed our previous results for T_reg_ cell expansion according to our GMP-compliant process from patients with end-stage LD or KD.

Finally, the manufacturing process that we set up has the important aspect of flexibility, which might be extremely useful to comply with different logistic and clinical settings. A 21-day expansion process can be demanding for small academic groups like ours. For this reason, we examined the possibility of fractionating the expansion in order to have a process more adaptable to the needs of the laboratory. On this point, our preliminary data from a single run suggested that it might still be possible to restore the expansion ability of T_reg_ cells after thawing the intermediate product (e.g., 14-day-expanding T_reg_ cells). Indeed, according to the expansion curves we obtained, a high number of cells on day 14 was available to be frozen as a master cell bank for future expansion. Furthermore, starting from cells frozen at this point of the expansion curve would allow the more rapid achievement of a clinically relevant number of cells in only 7 days, with all the logistical advantages of a shorter time period and an easily programmable production facility. This means that the timing of infusion can be adapted to different conditioning schemes and even to occasional deviations due to logistic and/or clinical problems during a clinical trial. Also, it would give the clinician the opportunity to plan ATMP administration at the optimal time based on the patient’s clinical progress in the context of the adaptive study design.

## Conclusions

In conclusion, our data clearly highlight the strengths and pitfalls as well as the flexibility of a robust ex vivo approach to obtain high numbers of GMP-grade T_reg_ cells from patients who are candidates for liver or kidney transplantation. These results pave the way for the design of clinical trials to test the clinical impact of T_reg_ cell-mediated therapy approaches for induction of tolerance in patients undergoing solid organ transplantation.

## Methods

### Prerequisites: equipment requirements and facility characteristics

Manufacturing and QC tests were performed at the Cell Factory, a GMP facility of the public hospital Fondazione IRCCS Ca’ Granda Ospedale Maggiore Policlinico in Milano, Italy. The facility was authorized to produce ATMPs for the first time in 2007 by the Italian Drug Agency (Agenzia Italiana del Farmaco—AIFA) in compliance with European GMP regulations [36], and it has maintained the certification uninterruptedly until today.

The facility is a fully controlled plant for ATMP manufacturing, whose characteristics have been previously described [48, 49]. Briefly, all manufacturing procedures were performed in a class A environment (class II type A2 biological safety cabinet/tissue culture hood; i.e., biosafety cabinet) with a class B surrounding environment. Microbial contamination was monitored using settle plates, volumetric active air sampling, and surface and operator sampling with contact plates. Continuous airborne particle monitoring was mostly performed in a class A environment; however, during critical steps, it was carried out in a class B environment using automatic particle counters.

### Process validation design

Based on the guidelines of the European Medicines Agency [50] for process design and validation, we performed the following actions: (1) set the quality target product profile and sampling plan to identify critical quality attributes for starting materials, intermediate product, and finished product; (2) collected prevalidation data from small-scale and scale-up experiments as well as in vivo studies; (3) identified the required equipment and facility characteristics; (4) defined an appropriate manufacturing process and identified a control strategy using process design and process risk analysis; (5) evaluated and confirmed the design process using established scientific evidence of the reproducibility of the process for process validation; (6) performed continuous process verification, which consisted of assuring that the production process remained under control during the entire period of routine production; (7) produced batch records; (8) validated the analytical methods; and (9) assured that the finished product met all quality attributes stated in the specifications.

### Process risk identification

We assessed the process-related risks according to international guidelines [36, 51] by performing PHA, as described in Additional file 1: Table S1. PHA started with the systematic identification of all potential hazards and accidental events that could interfere with the quality target product profile at any step of the process. The outcome of PHA provides the risk ranking for each combination of *SxOxD,* where S represents the severity of the consequences of failure, O represents the probability of the hazard occurrence, and D represents the probability of detection, which means the chance of detecting a failure before it occurs. The output of PHA was the identification of required hazard controls and follow-up actions thanks to the use of customized ranking scales as a guide. We performed the in-process risk analysis from the process design phase to the end of product development.

### Process validation and release criteria

For product validation, we predefined the quality target product profile and critical quality attributes for the intermediate and finished products, and every validation batch of finished product was manufactured accordingly. Each validation run was documented by a corresponding validation report. All predefined acceptance criteria and specifications as well as the respective QC results for starting materials, in-process intermediate, and finished product were recorded at every step of the manufacturing process. At the end of the process validation, a careful review of the predefined acceptance criteria for the quality target product profile of the starting materials and intermediate product was performed, in case changes needed to be made. The same procedure was applied for the release specifications of the finished product. The tests for batch release included cell count, purity, cell viability, and sterility (mycoplasma test and endotoxin quantification). Due to the nature of the expansion procedure, we included the quantification of residual anti-CD3/CD28-coated beads as part of the release tests. Details on the QC methods are provided below.

### Manufacturing process and in-process controls

#### Donor selection and starting material

The starting material was obtained from patients on the waiting list for solid-organ transplantation for LD or KD. Patient selection was based on the following inclusion criteria: (1) age ≥ 18 years; (2) diagnosis of end-stage KD and on the waiting list for a living-donor kidney transplant or the diagnosis of end-stage LD and on the waiting list for a liver transplant. Exclusion criteria included the following: (1) positivity by serology and nucleic acid testing (NAT) human immunodeficiency virus (HIV), hepatitis B virus (HBV), or hepatitis C virus (HCV) positivity; (2) syphilis antibody positivity; (3) combined transplant patients; (4) concurrent uncontrolled infection.

Steady-state leukapheresis was performed in two kidney transplant patients and two liver transplant patients at the clinical center of Azienda Ospedaliero-Universitaria di Bologna using a continuous-flow cell separator COM.TEC^®^ (Fresenius Kabi AG, Bad Homburg, Germany). Treatment with two blood volumes was set up as the procedure endpoint. Anticoagulant citrate dextrose solution, formula A (ACD-A) at a ratio of 1:14 to 1:13 was used to prevent coagulation. For prophylaxis of citrate-related hypocalcemia, calcium gluconate was administered intravenously during leukapheresis.

Each leukapheresis sample was transported at a controlled temperature (+ 4 to + 22 °C) to the GMP facility; continuous temperature recording was performed using a validated data logger (Testo Spa, Settimo Milanese, Italy). During validation the transportation temperature was recorded and used to set the range of acceptability to be used during the clinical trial. The sample was sent with the corresponding accompanying documentation, which included the same pertinent information as for the protocol. Sample collection and transport were set as follows: the duration of the collection procedure was set to be less than 4 h [52], and the leukapheresis product was to be received by the GMP facility within 4 h from the completion of sample collection. The leukapheresis sample was collected into prepared, sterile, and sealable bags, which were opened immediately before the sample was put in and closed as quickly as possible afterwards. The bag was appropriately sealed and identified by an anonymous univocal code. The other data reported on the leukapheresis label included the apheresis volume/weight, collection date, and duration of the harvesting procedure. In addition, the accompanying documentation reported the duration of transportation and temperature tracking.

Once approved as a starting material for the manufacturing process, sampling of the starting material was performed to check the cell count (ABX Micros 60 CT, HORIBA, Kyoto, Japan) and microbiological contamination using BacT/ALERT (bioMérieux, Marcy-l'Étoile, France). The contents of lymphocyte subpopulations were assessed as described below by flow cytometry; specifications for these parameters were set up at the end of three validation runs.

#### GMP-grade isolation of T_reg_ cells

The scheme for the entire manufacturing process of expanded T_reg_ cells is summarized in Fig. 1. Every step of the manufacturing process was documented and performed according to the batch records and standard procedures. Following the characterization of the finished product, the results were reviewed by the quality assurance group to define the release tests for the ATMP, as described in the process validation section.

CD8^−^ CD25^+^ cells were isolated from each leukapheresis sample (n = 4) under GMP conditions. Clinical-grade reagents and large-scale immunomagnetic cell separation systems (CliniMACS™ Instruments, Miltenyi Biotec, Bergisch Gladbach, Germany) were used in a two-step procedure, performed according to the manufacturer’s instructions, that included CD8^+^ T cell depletion followed by CD25^+^ T cell enrichment.

In detail, after drawing a representative sample to evaluate the number of T_reg_ cells by flow cytometry (see below), the leukapheresis product was transferred into the cell preparation bag and diluted at 1:3 with CliniMACS buffer (phosphate-buffered saline/EDTA buffer) supplemented with 0.5% human serum albumin (HSA/CliniMACS buffer). Cells were washed and resuspended in HSA/CliniMACS buffer in the indicated volume and labeled with CD8 MicroBeads (CliniMACS Miltenyi Biotec) for 30 min at room temperature on an orbital shaker. Unbound antibody was removed by washing, and cells were resuspended in 100 mL of HSA/CliniMACS buffer and depleted using the CliniMACS separation program “depletion 2.1”. The intermediate fraction (CD8^−^ cells) was collected and stored at 4 °C overnight.

For positive selection with anti-CD25 monoclonal antibody, cells were washed, resuspended in HSA/CliniMACS buffer, and incubated with CD25 microbeads (CliniMACS Miltenyi Biotec) for 15 min at 4–8 °C on an orbital shaker. After washing, the target cells were isolated using the CliniMACS separation program “enrichment 3.1.”

The positive fraction (CD8^−^ CD25^+^ cells) was collected, washed, and counted with an automated cell counter (Nucleocounter, Chemometech, Denmark, EU) to determine the number of total nucleated cells (TNCs). The purity of the isolated product was assessed by flow cytometry. Aliquots of positive and negative fractions (CD8^−^ CD25^−^ cells) were cryopreserved as described below.

To monitor the different isolation steps, different samples before and after each labeling, depletion, and enrichment step were analyzed by the QC laboratory to assess the purity and contaminants (flow cytometry) and the cell number (Nucleocounter). Negative fractions resulting from enrichment procedures, consisting of CD8^−^ CD25^−^ cells, were frozen in aliquots and cryopreserved for suppressive experiments in vitro.

#### GMP-grade expansion of T_reg_ cells

Clinical grade expansion was performed under GMP conditions in gas-permeable culture bags of increasing sizes (MACS GMP Cell Differentiation Bags, Miltenyi Biotec) for 3 weeks at 37 °C, 5% CO_2_, in complete medium consisting of TexMACS GMP medium supplemented with 100 nM rapamycin (MACS GMP Rapamycin, Miltenyi) and 5% allogeneic heat-inactivated plasma. In detail, 40 × 10^6^ isolated CD8^−^ CD25^+^ cells were seeded (day 0) at 0.5 × 10^6^ cells/mL in complete medium. The MACS GMP ExpAct T_reg_ Kit (MACS GMP ExpAct T_reg_ Beads conjugated to CD28 Anti-Biotin and CD3-Biotin monoclonal antibodies, Miltenyi Biotec) was used on days 0, 7, and 14 at different bead-to-cell ratios (4:1, 1:1, and 1:1, respectively). On day 2, 1000 IU/mL IL-2 (Proleukin, Novartis) was added. Cell feeding was performed every 2–3 days by doubling the culture volume through the addition of fresh medium supplemented with IL-2 at 1000 (days 5 and 7) or 500 IU/mL (days 9, 14, and 16). On days 7 and 14, cells were subcultured by seeding 1.0 × 10^6^ cells/mL in approximately 320 mL and 640 mL, respectively. At every medium feed starting from day 5, representative samples were tested with an automated cell counter (Nucleocounter, Chemometech, Denmark, EU), and the total cell number obtained was estimated based on the recorded sample-specific fold expansion. The immunophenotype was assessed on days 7, 14, and 21.

On day 21, bead removal was performed with magnetic selection using large-scale columns and the CliniMACS separation program “depletion 2.1,” according to the manufacturer’s instructions (Miltenyi Biotech). At the end of the procedure (day 21), expanded T_reg_ cells were collected after bead removal (target fraction), and a final and complete QC analysis was performed. The finished product consisted of the target fraction, which was cryopreserved in 20 mL of a solution composed of normal saline, human albumin (10% vol:vol), and dimethyl sulfoxide (DMSO; 10% vol:vol). To evaluate the possibility of restarting the culture from an intermediate product, cell expansion starting from thawed 14-day-expanded T_reg_ cells was performed (see the next paragraph).

#### Cryopreservation and thawing

Samples of freshly isolated CD8^−^ CD25^+^ cells (positive fraction) and CD8^−^ CD25^−^ cells (negative fraction) as well as expanded T_reg_ cells at different time points (days 14 and 21) were cryopreserved using a controlled-rate freezing system (Nicool Plus, Air Liquide, Paris, France) in a solution consisting of 80% sodium chloride (B. Braun, Melsungen, AG, Germany), 10% DMSO (CRYOSERV, Mylan Institutional, Inc., Canonsburg, PA, USA), and 10% HSA (Kedrion, Lucca, Italy) in cryobags (CryoMACS Freezing bag 50, Miltenyi Biotec). The frozen units were transferred and stored immediately in vapor-phase liquid nitrogen in dedicated tanks.

For functional and expansion tests, prewarmed thawing solution (80% sodium chloride, 10% HSA, and 3% ACD-A; Haemonetics) was added to freshly thawed cells; this condition allowed a higher T_reg_ cell viability and recovery among all the conditions tested in preliminary experiments (data not shown).

To evaluate the T_reg_ cell expansion ability after thawing, during the fourth validation run (#04), cell expansion was stopped at day 14, the cells were frozen and then thawed, and the cell viability and phenotype were assessed as mentioned above. The cell culture was restarted by seeding the cells in culture bag as described for the standard culture at the same time point.

### QCs: in-process and release tests

In-process QCs were performed on freshly expanding T_reg_ cells (days 7, 14, and 21). All release tests were performed on the finished products using the same general approach described previously [49]. Every CQ test was performed in duplicate to increase the data set.

#### Flow cytometry

The T_reg_ cell phenotype was determined by multi-color direct immunofluorescence with panels of monoclonal antibodies directed against the surface molecules CD4, CD8, CD19, CD25, CD45, CD56, and CD127 (all from BD Biosciences, San Jose, CA, USA) and the intracellular protein FoxP3 (FoxP3 Monoclonal Antibody, PE, eBioscience, San Diego, CA). For details on antibodies see Additional file 1: Table S2. For intracellular staining, 0.5 × 10^6^ cells were fixed and permeabilized using the eBioscience Foxp3/Transcription Factor Staining Buffer Set, according to the manufacturer’s instructions, and then stained with Foxp3 PE or Rat IgG2a kappa Isotype Control PE (eBioscience) as a negative control (Additional file 1: Fig. S1).

The T_reg_ cell immunophenotype, defined as CD45^+^ CD4^+^ CD25^+^ cells, was assumed to be the identity of the finished product [35]. The product purity was measured as the percentage of CD45, CD4, and CD25 co-expression; the contaminant populations were also analyzed “for information only” as the percentage of cells expressing CD8, CD19, and CD56. The expression of CD127 and FoxP3 on T_reg_ cells was also evaluated.

The cell viability was assayed by 7-aminoactinomycin D staining (BD Biosciences, San Jose, CA, USA). The viability after thawing the expanded T_reg_ cells was also evaluated “for information only.”

To measure the number of T_reg_ cells present in the leukapheresis and the respective fractions derived from the immunoselection procedure (starting material, postdepletion, and postselection samples), we used a single-platform technology based on an internal bead standard, a 6-color flow cytometer, and a sequential gating strategy to determine CD45^+^CD4^+^CD25^+^ absolute counts, similar to that described previously [53, 54]. Briefly, 100 μL of whole leukapheresis blood was transferred to Becton Dickinson (BD) Trucount tubes and stained as detailed in Additional file 1: Table S2. After staining, the erythrocytes were lysed for 10 min in ammonium chloride solution without washing. The Trucount tubes were then analyzed, and the number of cells/μL of whole blood was calculated in a similar manner to the previously described approach [53]. The T_reg_ cell gate was set using the CD25-minus-one control and represented as a percentage of CD45^+^ cells. The absolute number of T_reg_ cells/μL of whole blood was calculated according to the manufacturer’s equation: [number of events of interest/number of events] × [BD Trucount bead concentration/test volume in μL]. Cells were analyzed by a FACSCanto II cytometer (BD, Franklin Lakes, NJ, USA). To reduce variation in the setting gates, the standard operating procedures included example gates. Data were analyzed using Diva 8.0 software (BD).

#### Cell count

The cell count was validated according to an internal protocol and procedures [49]. Concerning the cell count, for every forty million cells seeded, we estimated that at least 1 × 10^9^ cells would be obtained postexpansion. We aimed to obtain > 1 × 10^9^ TNCs after removal of the culture beads.

#### Microbial contamination

Four different batches of 21-day-expanded T_reg_ cells were used to validate the methods used to detect microbial contamination (e.g., presence of aerobic and anaerobic bacteria, presence of endotoxins and mycoplasma), according to the European Pharmacopoeia (Ph. Eur.) using the validation strategy described previously [49]. The sterility tests were performed by a GMP- authorized external supplier.

#### Sterility

The test for sterility was carried out according to Ph. Eur. Chapter 2.6.27 (Edition 9.2) by direct inoculation of the microbial culture media with the sample product to be examined [55]. Method validation was performed to exclude any interference of the components of the cryopreserved finished product with microbial growth. For this purpose, the Ph. Eur.-recommended microbiological strains (ATCC Manassas, VA, USA) were inoculated in the finished product just before its cryopreservation, and microbial growth was assayed after thawing [49]. The lyophilized bacterial, yeast, and fungal strains were appropriately prepared and isolated in casein soya bean digest agar and Sabouraud, right-agar plates (Merck Millipore, MA, USA). Each batch of microbial culture medium was tested for sterility and fertility (growth promotion test). Three validation runs were performed by evaluating three different batches of 21-day-expanded T_reg_ cells (1% in volume of the finished product). Two levels of contamination were considered for each microorganism [1–10 colony-forming units (CFU) and 10–100 CFU]. As positive controls, control microorganisms were inoculated in complete medium without the finished product (T_reg_ cells); the finished product alone (without the microbial strains) was used as a negative control. The samples were incubated at 35–37 °C for 7 days. Negative controls were incubated at 35–37 °C for 14 days. The results were determined by visual observation of the colonies. The specificity, sensitivity, and intermediate precision were evaluated.

#### Bacterial endotoxin test analysis

The limulus amebocyte lysate (LAL) test for bacterial endotoxins was performed to fulfill the requirements for compliance with Ph. Eur. Chapter 2.6.14 method A (gel-clot method: limit test). For the endotoxin level, specification was defined at the end of the analytical method validation; the value was initially set at 5.8 endotoxin units (EU)/mL, which corresponds to the endotoxin limit for the product (Ph. Eur. 2.6.14).

The method was validated to determine the possible interference of the finished product (T_reg_ cells resuspended in a solution of normal saline, human albumin for human i.v. use (10% vol:vol), and DMSO (10% vol:vol)) in the clot formation by the Gel-Clot LAL test. The validation protocol consisted of the following four steps: (1) Confirmation of the labeled lysate sensitivity: LAL with a declared sensitivity (λ) of 0.03 EU/mL (Lonza, Walkersville, MD, USA) and a positive CSE (Lonza) were used. CSE, supplied in lyophilized form, was reconstituted with pyrogen-free water (water for a bacterial endotoxin test, Lonza) and serially diluted in the presence of 0.1 mL of LAL according to Ph. Eur. for the confirmation of λ. (2) Study of the product: Calculation of the endotoxin limit (EL) and the maximum valid dilution (MVD). According to Ph. Eur., the EL (EU/mL) was calculated by the formula K/M, where K is the threshold pyrogenic dose of endotoxin/kg of body mass (for intravenous/parenteral administration of 5.0 EU/kg) [39] and M is the maximum recommended dose of the product/kg of body mass (M is the maximum cellular dose (volume) infused/kg). The MVD value was calculated using the formula: MVD = EL/λ. (3) Preliminary test for interfering factors: this step was performed at various dilutions of the product (according to the MVD) in order to find the best dilution not activating and/or inhibiting the enzymatic reaction. The product, under the experimental conditions used, was determined not to contain interfering factors if the sensitivity of the lysate established with the diluted product in the presence of CSE was not less than 0.5λ and not greater than 2λ. (4) Test for interfering factors using the chosen dilution on three batches of product.

#### Mycoplasma

The culture-based mycoplasma test was validated on the finished product (T_reg_ cells resuspended in complete medium) and performed according to Ph. Eur. 2.6.7. The aim was to demonstrate that the cell culture supernatant (T_reg_ cells in complete medium, as described previously) does not contain substances that could interfere or inhibit the growth of mycoplasma. The reference strains of *Mycoplasma pneumoniae* (ATCC 15531) and *Mycoplasma hominis* (ATCC 23714) were transplanted on plates of Mycoplasma broth base (Millipore) and incubated at 35–38 °C for 7 days. After measuring the absorbance with a spectrophotometer (λ = 625), the suspension was diluted to obtain a concentration of 10–100 CFU/mL. The number of colonies of mycoplasma was determined by performing the count in double by inclusion in agar and incubating the plates at 35–38 °C. For the analysis, 10 mL and 0.2 mL of cell culture medium supernatant from three different productions were inoculated in liquid Hayflick medium and solid Hayflick medium, respectively (both from Merck Millipore). Mycoplasma strains were added at a concentration 10–100 CFU/mL. The samples were incubated for 21 days, and the number of CFU/mL was determined at 2–4 days, 6–8 days, 13–15 days, and 19–21 from the inoculums on the solid medium. The validation was confirmed if the following acceptance criteria were met: in the liquid medium, the growth of mycoplasma occurred simultaneously in the presence and in the absence of the product; and, in the solid medium, a difference greater than a factor of 5 was not observed in the CFU/mL determination between the inoculated plates in the presence and absence of the product.

#### Determination of residual MACS GMP ExpAct Beads in the finished product of T_reg_ cells

For determination of the residual beads in the finished product, three samples were considered: (1) positive control: 4 × 10^3^ beads without cells, obtained by serial dilution of the stock solution (0.2 × 10^6^ beads/µL); (2) original fraction: 3 × 10^6^ 21-day-expanded cells, before bead removal; (3) target fraction: 30 × 10^6^ 21-day-expanded cells, after bead immunodepletion. For the positive control and the original fraction, we tested an aliquot (10 µL) in a Bürker chamber to quantify the number of beads in the unprocessed sample.

Next, all samples (positive control, original fraction, and target fraction) were processed twice as follows: centrifugation (14,000 rpm, 5 min), lysis via the addition of 1 mL of 0.52% hypochlorite (30 s by vortexing), and centrifugation (14,000 rpm, 5 min). Finally, pellets were resuspended in phosphate-buffered saline to reach a final volume of 600 µL for the original fraction, 40 µL for the target fraction, and 200 µL for the positive control. Beads in a 40-μL sample were counted in a Bürker chamber by two different operators, twice each (10-µL aliquots, n = 4) by scanning for beads in the whole Bürker chamber. The recovery factor, the ratio between the number of beads in the lysed original fraction and the number of beads in the unprocessed original fraction, was calculated. The results of the Bürker counts were then adjusted with this recovery factor. The adjusted values from two operators were averaged and assumed to be the exact number of beads in 40 μL. The target fraction was resuspended in 40 μL. According to Miltenyi’s protocol, the specification was set as ≤ 1000 beads per 30 × 10^6^ cells.

### In vitro suppression assay

The suppressive function of T_reg_ cells in vitro was determined by a carboxyfluorescein diacetate succinimidyl ester (CFSE)-based suppression assay, as reported previously [7]. Briefly, cryopreserved autologous CD8^−^ CD25^−^ cells (used as T_eff_ cells) were thawed and labeled with 5 µM CFSE (Thermo Fischer Scientific, Carlsbad, CA, USA). T_eff_ cells were cocultured with thawed autologous expanded T_reg_ cells (day 21) at different T_reg_ cell:T_eff_ cell ratios (1:1 to 1:10). Cocultures were carried out in RPMI-1640 medium supplemented with 10% fetal bovine serum, 1% penicillin/streptomycin, and 1% l-glutamine in the presence of MACS GMP ExpAct T_reg_ kit reagents (bead:T_eff_ cell ratio: 1:10). On day 5, the cells were harvested and the residual amount of CFSE was quantified by flow cytometry using FlowJo software (Treestar); the proliferation of T_eff_ cells alone was taken as 100% proliferation.

### Statistical analyses

Statistical analyses were performed with GraphPad Prism 8 software (GraphPad Software, San Diego, CA, USA) using the appropriate tests. Unless otherwise noted, the data from KD and LD patients were compared using the Student’s t test or two-way analysis of variance for multiple comparisons. Numerical data are presented as the median and range or mean ± standard deviation, as appropriate. p values < 0.05 were considered statistically significant.

## Supplementary Information

**Additional file 1.** **Supplementary table 1**. Criticality matrices for PHA. **Supplementary table 2**. Multicolor flow cytometry for investigational medicinal product characterization: type and quantity of antibodies per assessment. **Supplementary figure 1**. Negative control for FoxP3 staining

## Data Availability

The datasets generated and/or analyzed in the current study are available from the corresponding author on reasonable request.

## References

[1] Pilat N, Sprent J (2021). Treg therapies revisited: tolerance beyond deletion. Front Immunol.

[2] Que W, Li X-K. Regulatory T cells for the induction of transplantation tolerance. Adv Exp Med Biol. 2021; 289–302. 10.1007/978-981-15-6407-9_15 33523454

[3] Romano M, Fanelli G, Albany CJ, Giganti G, Lombardi G (2019). Past, present, and future of regulatory T cell therapy in transplantation and autoimmunity. Front Immunol.

[4] Joffre O, Santolaria T, Calise D, Al ST, Hudrisier D, Romagnoli P (2008). Prevention of acute and chronic allograft rejection with CD4 +CD25+Foxp3+ regulatory T lymphocytes. Nat Med.

[5] Golshayan D, Jiang S, Tsang J, Garin MI, Mottet C, Lechler RI (2007). In vitro-expanded donor alloantigen-specific CD4+CD25+ regulatory T cells promote experimental transplantation tolerance. Blood.

[6] Taylor PA, Panoskaltsis-Mortari A, Swedin JM, Lucas PJ, Gress RE, Levine BL (2004). L-selectinhi but not the L-selectinlo CD4 +25+ T-regulatory cells are potent inhibitors of GVHD and BM graft rejection. Blood.

[7] Ulbar F, Montemurro T, Jofra T, Capri M, Comai G, Bertuzzo V (2019). Regulatory T cells from patients with end-stage organ disease can be isolated, expanded and cryopreserved according good manufacturing practice improving their function. J Transl Med.

[8] Di Ianni M, Falzetti F, Carotti A, Terenzi A, Castellino F, Bonifacio E (2011). Tregs prevent GVHD and promote immune reconstitution in HLA-haploidentical transplantation. Blood.

[9] Search of: expanded t reg—List Results—ClinicalTrials.gov [Internet]. [cited 2021 Apr 9]. Available from: https://clinicaltrials.gov/ct2/results?recrs=&cond=&term=expanded+t+reg&cntry=&state=&city=&dist=.

[10] Lee K, Nguyen V, Lee KM, Kang SM, Tang Q (2014). Attenuation of donor-reactive T cells allows effective control of allograft rejection using regulatory T cell therapy. Am J Transplant.

[11] Zhang H, Guo H, Lu L, Zahorchak AF, Wiseman RW, Raimondi G (2015). Sequential monitoring and stability of ex vivo -expanded autologous and nonautologous regulatory T cells following infusion in nonhuman primates. Am J Transplant.

[12] Brunstein CG, Miller JS, Cao Q, McKenna DH, Hippen KL, Curtsinger J (2011). Infusion of ex vivo expanded T regulatory cells in adults transplanted with umbilical cord blood: safety profile and detection kinetics. Blood.

[13] Pigeau GM, Csaszar E, Dulgar-Tulloch A (2018). Commercial scale manufacturing of allogeneic cell therapy [Internet]. Front Med.

[14] Guo W-W, Su X-H, Wang M-Y, Han M-Z, Feng X-M, Jiang E-L (2021). Regulatory T cells in GVHD therapy. Front Immunol.

[15] MacDonald KN, Piret JM, Levings MK (2019). Methods to manufacture regulatory T cells for cell therapy. Clin Exp Immunol.

[16] Fan Z, Spencer JA, Lu Y, Pitsillides CM, Singh G, Kim P (2010). In vivo tracking of “color-coded” effector, natural and induced regulatory T cells in the allograft response. Nat Med.

[17] Tang Q, Lee K. Regulatory T-cell therapy for transplantation: how many cells do we need?. Curr Opin Organ Transplant. 2012; 349–54. 10.1097/MOT.0b013e328355a992 22790069

[18] Ezzelarab MB, Thomson AW (2016). Adoptive cell therapy with Tregs to improve transplant outcomes: the promise and the stumbling blocks. Curr Transplant Reports..

[19] Ng WF, Duggan PJ, Ponchel F, Matarese G, Lombardi G, David Edwards A (2001). Human CD4+CD25+ cells: a naturally occurring population of regulatory T cells. Blood.

[20] Alsuliman A, Appel SH, Beers DR, Basar R, Shaim H, Kaur I (2016). A robust, good manufacturing practice–compliant, clinical-scale procedure to generate regulatory T cells from patients with amyotrophic lateral sclerosis for adoptive cell therapy. Cytotherapy.

[21] Wiesinger M, Stoica D, Roessner S, Lorenz C, Fischer A, Atreya R, et al. Good manufacturing practice-compliant production and lot-release of ex vivo expanded regulatory T cells as basis for treatment of patients with autoimmune and inflammatory disorders. Front Immunol [Internet]. 2017;8. 10.3389/fimmu.2017.01371 PMC5662555 29123521

[22] Voskens CJ, Fischer A, Roessner S, Lorenz C, Hirschmann S, Atreya R (2017). Characterization and expansion of autologous GMP-ready regulatory T cells for TREG-based cell therapy in patients with ulcerative colitis. Inflamm Bowel Dis.

[23] Seay HR, Putnam AL, Cserny J, Posgai AL, Rosenau EH, Wingard JR (2017). Expansion of human Tregs from cryopreserved umbilical cord blood for GMP-compliant autologous adoptive cell transfer therapy. Mol Ther Methods Clin Dev [Internet]..

[24] Battaglia M, Stabilini A, Roncarolo MG (2005). Rapamycin selectively expands CD4+CD25+FoxP3 + regulatory T cells. Blood.

[25] Fraser H, Safinia N, Grageda N, Thirkell S, Lowe K, Fry LJ (2018). A Rapamycin-based GMP-compatible process for the isolation and expansion of regulatory T cells for clinical trials. Mol Ther Methods Clin Dev.

[26] REGULATION (EC) No 1394/2007 OF THE EUROPEAN PARLIAMENT AND OF THE COUNCIL of 13 November 2007 on advanced therapy medicinal products and amending Directive 2001/83/EC and Regulation (EC) No 726/2004 (Text with EEA relevance). REGULATION (EC) No 1394/2007 OF THE EUROPEAN PARLIAMENT AND OF THE COUNCIL of 13 November 2007 on advanced therapy medicinal products and amending Directive 2001/83/EC and Regulation (EC) No 726/2004 (Text with EEA relevance).

[27] Garakani R, Saidi RF (2017). Recent progress in cell therapy in solid organ transplantation. Int J Organ Transplant Med [Internet]..

[28] Safinia N, Vaikunthanathan T, Fraser H, Thirkell S, Lowe K, Blackmore L (2016). Successful expansion of functional and stable regulatory T cells for immunotherapy in liver transplantation. Oncotarget.

[29] Afzali B, Edozie FC, Fazekasova H, Scottà C, Mitchell PJ, Canavan JB (2013). Comparison of regulatory T cells in hemodialysis patients and healthy controls: implications for cell therapy in transplantation. Clin J Am Soc Nephrol.

[30] McDonald-Hyman C, Turka LA, Blazar BR. Advances and challenges in immunotherapy for solid organ and hematopoietic stem cell transplantation [Internet]. Sci Transl Med. 2015. Available from: https://pubmed.ncbi.nlm.nih.gov/25810312/. 10.1126/scitranslmed.aaa6853 PMC4425354 25810312

[31] Ukena SN, Höpting M, Velaga S, Ivanyi P, Grosse J, Baron U (2011). Isolation strategies of regulatory T cells for clinical trials: phenotype, function, stability, and expansion capacity. Exp Hematol.

[32] Velaga S, Alter C, Dringenberg U, Thiesler CT, Kuhs S, Olek S (2017). Clinical-grade regulatory T cells: comparative analysis of large-scale expansion conditions. Exp Hematol [Internet]..

[33] McKenna DH, Sumstad D, Kadidlo DM, Batdorf B, Lord CJ, Merkel SC (2017). Optimization of cGMP purification and expansion of umbilical cord blood–derived T-regulatory cells in support of first-in-human clinical trials. Cytotherapy.

[34] Di Ianni M, Del Papa B, Zei T, Iacucci Ostini R, Cecchini D, Cantelmi MG, et al. T regulatory cell separation for clinical application [Internet]. Transfus Apher Sci 2012; 213–6. 10.1016/j.transci.2012.06.007 22795999

[35] Gołąb K, Krzystyniak A, Marek-Trzonkowska N, Misawa R, Wang LJ, Wang X (2013). Impact of culture medium on CD4+ CD25highCD127lo/neg Treg expansion for the purpose of clinical application. Int Immunopharmacol.

[36] EudraLex. EudraLex—Volume 4—Good Manufacturing Practice (GMP) guidelines | Public Health [Internet]. EudraLex—Vol. 4—Good Manuf. Pract. Guidel. 2003 [cited 2021 Apr 8]. Available from: https://ec.europa.eu/health/documents/eudralex/vol-4_en.

[37] Good distribution practice | European Medicines Agency [Internet]. [cited 2021 Oct 21]. Available from: https://www.ema.europa.eu/en/human-regulatory/post-authorisation/compliance/good-distribution-practice.

[38] Hori S, Nomura T, Sakaguchi S (2003). Control of regulatory T cell development by the transcription factor Foxp3. Science.

[39] Wachtel RE, Tsuji K (1977). Comparison of limulus amebocyte lysates and correlation with the United States Pharmacopeial pyrogen test. Appl Environ Microbiol.

[40] Peters JH, Preijers FW, Woestenenk R, Hilbrands LB, Koenen HJPM, Joosten I (2008). Clinical grade Treg: GMP isolation, improvement of purity by CD127pos depletion, Treg expansion, and Treg cryopreservation. PLoS ONE.

[41] Florek M, Schneidawind D, Pierini A, Baker J, Armstrong R, Pan Y (2015). Freeze and Thaw of CD4+CD25+Foxp3+ regulatory T cells results in loss of CD62L expression and a reduced capacity to protect against graft-versus-host disease. PLoS One [Internet]..

[42] Golab K, Leveson-Gower D, Wang X-J, Grzanka J, Marek-Trzonkowska N, Krzystyniak A (2013). Challenges in cryopreservation of regulatory T cells (Tregs) for clinical therapeutic applications. Int Immunopharmacol.

[43] Sánchez-Fueyo A, Whitehouse G, Grageda N, Cramp ME, Lim TY, Romano M (2020). Applicability, safety, and biological activity of regulatory T cell therapy in liver transplantation. Am J Transplant [Internet]..

[44] Todo S, Yamashita K, Goto R, Zaitsu M, Nagatsu A, Oura T (2016). A pilot study of operational tolerance with a regulatory T-cell-based cell therapy in living donor liver transplantation. Hepatology [Internet]..

[45] miltenyi biotech. (No Title) [Internet]. [cited 2021 Apr 8]. Available from: https://www.miltenyibiotec.com/_Resources/Persistent/c6a0b41674efcebbb1a3243d5b38a740a996d017/IM0017462.pdf.

[46] Haase D, Puan KJ, Starke M, Lai TS, Soh MYL, Karunanithi I (2015). Large-scale isolation of highly pure “untouched” regulatory T cells in a GMP environment for adoptive cell therapy. J Immunother [Internet]..

[47] Hoffmann P, Boeld TJ, Eder R, Albrecht J, Doser K, Piseshka B (2006). Isolation of CD4+CD25+ regulatory T cells for clinical trials. Biol Blood Marrow Transplant [Internet]..

[48] Montemurro T, Viganò M, Budelli S, Montelatici E, Lavazza C, Marino L (2015). How we make cell therapy in Italy. Drug Des Devel Ther [Internet]..

[49] Viganò M, Budelli S, Lavazza C, Montemurro T, Montelatici E, de Cesare S (2018). Tips and tricks for validation of quality control analytical methods in good manufacturing practice mesenchymal stromal cell production. Stem Cells Int [Internet]..

[50] Agency EM. Guideline on process validation for finished products—information and data to be provided in regulatory submissions [Internet]. 2014 [cited 2021 Apr 8]. p. 1–15. Available from: https://www.ema.europa.eu/en/process-validation-finished-products-information-data-be-provided-regulatory-submissions.

[51] ICH Q9 Quality risk management | European Medicines Agency [Internet]. [cited 2021 Apr 19]. Available from: https://www.ema.europa.eu/en/ich-q9-quality-risk-management.

[52] Cassens U, Barth IM, Baumann C, Fischer RJ, Kienast J, Vormoor J (2004). Factors affecting the efficacy of peripheral blood progenitor cells collections by large-volume leukaphereses with standardized processing volumes. Transfusion.

[53] Hardy MY, Vari F, Rossetti T, Hart DN, Prue RL (2013). A flow cytometry based assay for the enumeration of regulatory T cells in whole blood. J Immunol Methods.

[54] Hensley TR, Easter AB, Gerdts SE, De Rosa SC, Heit A, McElrath MJ, et al. Enumeration of major peripheral blood leukocyte populations for multicenter clinical trials using a whole blood phenotyping assay. J Vis Exp. 2012;e4302. Available from: https://pubmed.ncbi.nlm.nih.gov/23007739/. 10.3791/4302 PMC3490252 23007739

[55] European Pharmacopoeia. European Pharmacopoeia—Chapter 2.6.27 Microbiological Examination of cell-based Preparations revised—ECA Academy [Internet]. [cited 2021 Apr 9]. Available from: https://www.gmp-compliance.org/gmp-news/european-pharmacopoeia-chapter-2-6-27-microbiological-examination-of-cell-based-preparations-revised.

[56] BioRender [Internet]. [cited 2021 Apr 9]. Available from: https://biorender.com/.

