# Supplementary material for: Process development and validation of expanded regulatory T cells for prospective applications: an example of manufacturing a personalized advanced therapy medicinal product
Source: J Transl Med. 2022 Jan 5;20:14. doi: 10.1186/s12967-021-03200-x (PMC8729072; doi:10.1186/s12967-021-03200-x)
Supplement: Supplementary file 1 — Additional file 1. Supplementary table 1. Criticality matrices for PHA. Supplementary table 2. Multicolor flow cytometry for investigational medicinal product characterization: type and quantity of antibodies per assessment. Supplementary figure 1. Negative control for FoxP3 staining [file 12967_2021_3200_MOESM1_ESM.docx]

Supplementary table 1. Criticality matrices for PHA.

|  |  | **Occurrence** | | | |  |  |
| --- | --- | --- | --- | --- | --- | --- | --- |
|  |  | **1** | **2** | **3** | **4** |  |  |
| **Severity** | **1** | 1 | 2 | 3 | 4 | **1** | **Detection** |
|  | **2** | 4 | 8 | 12 | 16 | **2** |  |
|  | **3** | 9 | 18 | 27 | 36 | **3** |  |
|  | **4** | 16 | 32 | 48 | 64 | **4** |  |

| **CRITICALITY SCALE** | | |
| --- | --- | --- |
| **Index** | **Level of criticality** | **Decision** |
| 1-17 | Acceptable | No action required |
| 18-35 | Tolerable | Appropriate management should be implemented |
| 36-64 | Unacceptable | Mitigation measures must be implemented |

| **SCALE USED FOR ESTIMATION OF SCORE ASSOCIATED WITH EACH IDENTIFIED RISK** | | | | | |
| --- | --- | --- | --- | --- | --- |
| **SEVERITY** | | | **LIKELIHOOD** | | |
| **score** | **Impact on GMP product quality**  **(S)** | | **Score** | **Probability of occurrence**  **(O)** | **Probability of Detection**  **(D)** |
| 1 | Minor | No significant impact on CQA | 1 | Remote event | Event frequently detectable / before CQ test conclusion |
| 2 | Major | Minimal and reversible impact on CQA | 2 | Occasional event | event easily detectable with appropriate control system |
| 3 | Critical | Critically alter CQA (potential regulatory incompliance) | 3 | Frequent event | Event for which control systems can identify failures/ after CQ conclusion |
| 4 | Catastrophic | Highly alter the product safety / need for recall(regulatory incompliance) | 4 | Very frequent event | Undetectable event |

CQA, critical quality attributes.

Supplementary table 2. Multicolor flow cytometry for investigational medicinal product characterization: type and quantity of antibodies per assessment.

|  | **Antibody staining panel to assess:** | | | | **T_reg_ cells enumeration [Trucount]** | **FoxP3**  **expression** | **Contami-nating cells** |
| --- | --- | --- | --- | --- | --- | --- | --- |
|  | CD4 | FITC | BD Multi-Clone | SK3, SK4 | 20 µL | 20 µL | 20 µL |
|  | CD8 | PE | BD Pharmingen | HIT8a | 20 µL | / | / |
|  | IgG2a kappa  *or*  FoxP3 | PE  PE | eBioscience  eBioscience | eBR2a  PCH101 | / | 0.5 µL  5 µL | / |
|  | CD56 | PE | BD Pharmingen | B159 | / | / | 5 µL |
|  | 7-AAD | / | BD | / | 20 µL | 20 µL | 20 µL |
|  | CD127 | PE-Cy7 | BD Pharmingen | HIL-7R-M21 | 5 µL | 5 µL | / |
|  | CD19 | PE-Cy7 | BD Pharmingen | HIB19 | / | / | 5 µL |
|  | CD25 | APC | BD Pharmingen | M-A251 | 20 µL | 20 µL | / |
|  | CD8 | APC | BD Pharmingen | M-A251 | / | / | 20 µL |
|  | CD45 | APC-H7 | BD Pharmingen | 2D1 | 5 µL | 5 µL | 5 µL |

Supplementary figure 1. Negative control for FoxP3 staining.

For assessment of FoxP3 expression on T_reg_ cells, postenrichment and postexpansion samples were stained with Foxp3 PE or the corresponding isotype control, and analyzed according to the gating strategy described in Figure 2. A, B: Plots displaying IgG2a kappa isotype control staining of postenrichment (A) and postexpansion (B) samples. C, D: Overlay of the isotype-stained control (blue) onto the corresponding FoxP3-stained sample (red, inset) for postenrichment (C) and postexpansion (D) samples. Representative images of the samples from patient KD2 depicted in Figure 2 are shown.
